# 2-Chloro-5-nitro­pyridin-4-amine

**DOI:** 10.1107/S1600536812017643

**Published:** 2012-04-28

**Authors:** Jian-Ling He

**Affiliations:** a College of Chemical and Biological Engineering, Yancheng Institute of Technology, Yinbing Road No.9 Yancheng, Yancheng 224051, People’s Republic of China

## Abstract

The title mol­ecule, C_5_H_4_ClN_3_O_2_, possesses mirror symmetry, with all of the atoms lying in the mirror plane. There is an intra­molecular N—H⋯O hydrogen bond involving the adjacent –NO_2_ and –NH_2_ groups. A short C—H⋯O inter­action is also observed. In the crystal, adjacent mol­ecules are linked *via* N—H⋯Cl and N—H⋯N hydrogen bonds, forming chains propagating along [100].

## Related literature

For details concerning the importance of the title compound as an inter­mediate in organic synthesis, and for the synthetic procedure, see: Hu *et al.* (2011 ▶). For bond-length data, see: Allen *et al.* (1987 ▶). 
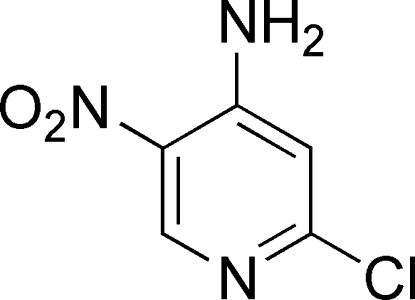


## Experimental

### 

#### Crystal data

C_5_H_4_ClN_3_O_2_
*M* *_r_* = 173.5
Orthorhombic, 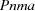

*a* = 14.596 (2) Å
*b* = 6.2782 (10) Å
*c* = 7.3018 (12) Å
*V* = 669.11 (18) Å^3^
*Z* = 4
Mo *K*α radiation
μ = 0.52 mm^−1^
*T* = 296 K
0.18 × 0.17 × 0.15 mm

#### Data collection

Enraf–Nonius CAD-4 diffractometer
Absorption correction: multi-scan (North *et al.*, 1968 ▶) *T*_min_ = 0.913, *T*_max_ = 0.927
3496 measured reflections
663 independent reflections
625 reflections with *I* > 2σ(*I*)
*R*_int_ = 0.034
3 standard reflections every 200 reflections intensity decay: 1%

#### Refinement

*R*[*F* ^2^ > 2σ(*F* ^2^)] = 0.028
*wR*(*F* ^2^) = 0.082
*S* = 1.16
663 reflections
67 parameters
H-atom parameters constrained
Δρ_max_ = 0.21 e Å^−3^
Δρ_min_ = −0.35 e Å^−3^

### 

Data collection: *CAD-4 Software* (Enraf–Nonius, 1985 ▶); cell refinement: *CAD-4 Software*; data reduction: *XCAD4* (Harms & Wocadlo, 1995 ▶); program(s) used to solve structure: *SHELXS97* (Sheldrick, 2008 ▶); program(s) used to refine structure: *SHELXL97* (Sheldrick, 2008 ▶); molecular graphics: *SHELXTL* (Sheldrick, 2008 ▶); software used to prepare material for publication: *SHELXTL*.

## Supplementary Material

Crystal structure: contains datablock(s) I, global. DOI: 10.1107/S1600536812017643/su2408sup1.cif

Structure factors: contains datablock(s) I. DOI: 10.1107/S1600536812017643/su2408Isup2.hkl

Supplementary material file. DOI: 10.1107/S1600536812017643/su2408Isup3.cml

Additional supplementary materials:  crystallographic information; 3D view; checkCIF report

## Figures and Tables

**Table 1:** Hydrogen-bond geometry (Å, °)

*D*—H⋯*A*	*D*—H	H⋯*A*	*D*⋯*A*	*D*—H⋯*A*
N2—H2*B*⋯O2	0.85	2.06	2.673 (3)	128
C5—H5⋯O1	0.93	2.34	2.682 (2)	101
N2—H2*A*⋯Cl1^i^	0.80	2.77	3.3023 (18)	126
N2—H2*B*⋯N1^i^	0.85	2.61	3.213 (2)	128

Symmetry code: (i) 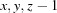.

**Fig. 1.**
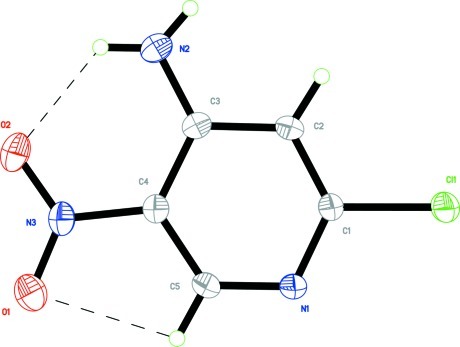
The molecular structure of the title molecule, with the atom-numbering. Displacement ellipsoids are drawn at the 50% probability level.

**Fig. 2.**
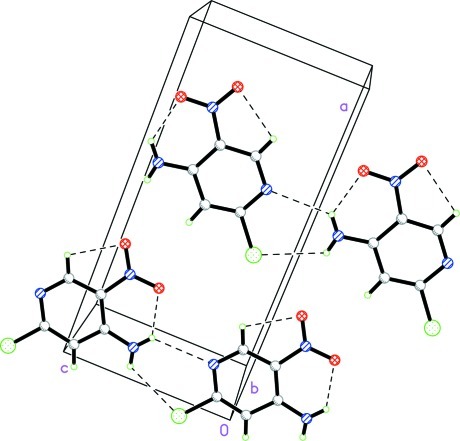
A view along the b axis of the crystal packing of the title compound. The hydrogen bonds are shown as dashed lines [H atoms not involved in hydrogen bonding have been omitted for clarity].

## supplementary crystallographic information

### Comment

The title compound is an important nitropyridine compound which is widely used in organic synthesis, especially in the synthesis of heterocyclic drugs and cytokine inhibitors (Hu *et al.*, 2011).

The molecular structure of the title compound is shown in Fig. 1. The molecule lies in a mirror plane. In the molecule there is an N-H···O hydrogen bond involving the adjacent NO_2_ and NH_2_ groups (Table 1). A short C-H···O interaction is also observed. The bond lengths (Allen *et al.*, 1987) and angles are within normal ranges.

In the crystal, adjacent molecules are linked via N–H···Cl and N–H···N hydrogen bonds so forming chains propagating along the a axis direction. (Table 1 and Fig. 2).

### Experimental

The title compound was prepared by the literature procedure (Hu *et al.*, 2011). To a solution of *tert*-butyl 2-chloro-5-nitropyridin-4-ylcarbamate (5 g, 18.3 mmol) in dichloromethane (30 ml) in a 100 mL flask was added slowly a solution of trifluoroaceticacid (10 ml). After being stirred for 4 h at the room temperature, the solvent was evaporated on a rotary evaporator. The pH of the remaining mixture was then adjusted to 7 with saturated sodium bicarbonate solution, giving the title compound. Colourless block-like crytsals were grown in ethanol (30 ml) by evaporating the solvent slowly at room temperature for about 8 d.

### Refinement

The NH H atoms were located in a difference Fourier map and were treated as riding atoms. The C-bound H-atoms were included in calculated positions and treated as riding atoms: C-H = 0.93 Å. For all H atoms U_iso_(H) = 1.2U_eq_(N,C).

### Crystal data

**Table:**

C_5_H_4_ClN_3_O_2_	*F*(000) = 352
*M**_r_* = 173.5	*D*_x_ = 1.723 Mg m^−^^3^
Orthorhombic, *P**n**m**a*	Mo *K*α radiation, λ = 0.71073 Å
Hall symbol: -P 2ac 2n	Cell parameters from 2718 reflections
*a* = 14.596 (2) Å	θ = 2.8–29.8°
*b* = 6.2782 (10) Å	µ = 0.52 mm^−^^1^
*c* = 7.3018 (12) Å	*T* = 296 K
*V* = 669.11 (18) Å^3^	Block, colourless
*Z* = 4	0.18 × 0.17 × 0.15 mm

### Data collection

**Table:**

Enraf–Nonius CAD-4 diffractometer	625 reflections with *I* > 2σ(*I*)
Radiation source: fine-focus sealed tube	*R*_int_ = 0.034
Graphite monochromator	θ_max_ = 25.2°, θ_min_ = 2.8°
ω/2θ scans	*h* = −17→17
Absorption correction: multi-scan (North *et al.*, 1968)	*k* = −6→7
*T*_min_ = 0.913, *T*_max_ = 0.927	*l* = −8→7
3496 measured reflections	3 standard reflections every 200 reflections
663 independent reflections	intensity decay: 1%

### Refinement

**Table:**

Refinement on *F*^2^	Primary atom site location: structure-invariant direct methods
Least-squares matrix: full	Secondary atom site location: difference Fourier map
*R*[*F*^2^ > 2σ(*F*^2^)] = 0.028	Hydrogen site location: inferred from neighbouring sites
*wR*(*F*^2^) = 0.082	H-atom parameters constrained
*S* = 1.16	*w* = 1/[σ^2^(*F*_o_^2^) + (0.0499*P*)^2^ + 0.1012*P*] where *P* = (*F*_o_^2^ + 2*F*_c_^2^)/3
663 reflections	(Δ/σ)_max_ < 0.001
67 parameters	Δρ_max_ = 0.21 e Å^−^^3^
0 restraints	Δρ_min_ = −0.35 e Å^−^^3^

### Special details

**Table:**

Geometry. All e.s.d.'s (except the e.s.d. in the dihedral angle between two l.s. planes) are estimated using the full covariance matrix. The cell e.s.d.'s are taken into account individually in the estimation of e.s.d.'s in distances, angles and torsion angles; correlations between e.s.d.'s in cell parameters are only used when they are defined by crystal symmetry. An approximate (isotropic) treatment of cell e.s.d.'s is used for estimating e.s.d.'s involving l.s. planes.
Refinement. Refinement of *F*^2^ against ALL reflections. The weighted *R*-factor *wR* and goodness of fit *S* are based on *F*^2^, conventional *R*-factors *R* are based on *F*, with *F* set to zero for negative *F*^2^. The threshold expression of *F*^2^ > σ(*F*^2^) is used only for calculating *R*-factors(gt) *etc*. and is not relevant to the choice of reflections for refinement. *R*-factors based on *F*^2^ are statistically about twice as large as those based on *F*, and *R*- factors based on ALL data will be even larger.

### Fractional atomic coordinates and isotropic or equivalent isotropic displacement parameters (Å^2^)

**Table:**

	*x*	*y*	*z*	*U*_iso_*/*U*_eq_	
Cl1	0.49841 (3)	0.2500	0.77100 (7)	0.0475 (2)	
O1	0.10325 (10)	0.2500	0.3741 (2)	0.0610 (5)	
O2	0.18179 (10)	0.2500	0.12421 (19)	0.0495 (4)	
N1	0.32242 (10)	0.2500	0.70422 (19)	0.0329 (4)	
N2	0.36482 (14)	0.2500	0.1360 (2)	0.0492 (5)	
H2A	0.4186	0.2500	0.1145	0.059*	
H2B	0.3196	0.2500	0.0623	0.059*	
N3	0.17674 (11)	0.2500	0.2932 (2)	0.0377 (4)	
C1	0.40493 (11)	0.2500	0.6229 (2)	0.0317 (4)	
C2	0.42218 (12)	0.2500	0.4395 (2)	0.0350 (4)	
H2	0.4821	0.2500	0.3964	0.042*	
C3	0.34767 (13)	0.2500	0.3155 (3)	0.0329 (4)	
C4	0.26029 (11)	0.2500	0.3989 (2)	0.0311 (4)	
C5	0.25209 (11)	0.2500	0.5895 (2)	0.0330 (4)	
H5	0.1934	0.2500	0.6392	0.040*	

### Atomic displacement parameters (Å^2^)

**Table:**

	*U*^11^	*U*^22^	*U*^33^	*U*^12^	*U*^13^	*U*^23^
Cl1	0.0280 (3)	0.0797 (4)	0.0347 (4)	0.000	−0.00544 (16)	0.000
O1	0.0275 (8)	0.0978 (12)	0.0575 (10)	0.000	−0.0049 (7)	0.000
O2	0.0551 (9)	0.0551 (8)	0.0384 (8)	0.000	−0.0163 (7)	0.000
N1	0.0277 (8)	0.0446 (8)	0.0264 (8)	0.000	0.0025 (5)	0.000
N2	0.0451 (10)	0.0759 (12)	0.0266 (9)	0.000	0.0030 (7)	0.000
N3	0.0346 (9)	0.0395 (8)	0.0390 (10)	0.000	−0.0097 (7)	0.000
C1	0.0261 (8)	0.0403 (9)	0.0288 (8)	0.000	−0.0020 (6)	0.000
C2	0.0262 (8)	0.0479 (10)	0.0310 (9)	0.000	0.0059 (7)	0.000
C3	0.0359 (10)	0.0355 (8)	0.0272 (8)	0.000	0.0020 (7)	0.000
C4	0.0296 (9)	0.0327 (8)	0.0311 (9)	0.000	−0.0026 (7)	0.000
C5	0.0258 (9)	0.0397 (9)	0.0333 (10)	0.000	0.0046 (6)	0.000

### Geometric parameters (Å, º)

**Table:**

Cl1—C1	1.7410 (16)	N3—C4	1.443 (2)
O1—N3	1.225 (2)	C1—C2	1.363 (2)
O2—N3	1.236 (2)	C2—C3	1.415 (3)
N1—C5	1.325 (2)	C2—H2	0.9300
N1—C1	1.343 (2)	C3—C4	1.413 (2)
N2—C3	1.335 (3)	C4—C5	1.397 (3)
N2—H2A	0.8009	C5—H5	0.9300
N2—H2B	0.8515		
			
C5—N1—C1	114.55 (14)	C1—C2—H2	120.4
C3—N2—H2A	112.1	C3—C2—H2	120.4
C3—N2—H2B	118.4	N2—C3—C4	126.34 (18)
H2A—N2—H2B	129.5	N2—C3—C2	118.97 (16)
O1—N3—O2	122.27 (16)	C4—C3—C2	114.70 (16)
O1—N3—C4	118.81 (16)	C5—C4—C3	120.44 (16)
O2—N3—C4	118.92 (16)	C5—C4—N3	117.42 (15)
N1—C1—C2	126.90 (15)	C3—C4—N3	122.14 (17)
N1—C1—Cl1	115.35 (12)	N1—C5—C4	124.29 (14)
C2—C1—Cl1	117.75 (13)	N1—C5—H5	117.9
C1—C2—C3	119.13 (16)	C4—C5—H5	117.9

### Hydrogen-bond geometry (Å, º)

**Table:**

*D*—H···*A*	*D*—H	H···*A*	*D*···*A*	*D*—H···*A*
N2—H2*B*···O2	0.85	2.06	2.673 (3)	128
C5—H5···O1	0.93	2.34	2.682 (2)	101
N2—H2*A*···Cl1^i^	0.80	2.77	3.3023 (18)	126
N2—H2*B*···N1^i^	0.85	2.61	3.213 (2)	128

Symmetry code: (i) *x*, *y*, *z*−1.
